# Mangiferin Facilitates Islet Regeneration and β-Cell Proliferation through Upregulation of Cell Cycle and β-Cell Regeneration Regulators

**DOI:** 10.3390/ijms15059016

**Published:** 2014-05-20

**Authors:** Hai-Lian Wang, Chun-Yang Li, Bin Zhang, Yuan-De Liu, Bang-Min Lu, Zheng Shi, Na An, Liang-Kai Zhao, Jing-Jing Zhang, Jin-Ku Bao, Yi Wang

**Affiliations:** 1 School of Life Sciences & Key Laboratory of Bio-Resources, Ministry of Education, Sichuan University, Chengdu 610064, China; E-Mails: hailianwang322@hotmail.com (H.-L.W.); vivian-goldenblue@163.com (C.-Y.L.); binzhang@163.com (B.Z.); lubangmin@126.com (B.-M.L.); drshiz1002@hotmail.com (Z.S.); anna1208lucky@gmail.com (N.A.); kailiangzhao@163.com (L.-K.Z.); 2 Institute of Organ Transplantation, Sichuan Academy of Medical Science & Sichuan Provincial People’s Hospital, Chengdu 610072, China; 3 Department of Pharmacy, 91889 Military Hospital, Zhanjiang 524001, China; E-Mail: yuandeliu@126.com; 4 Institute of Neurology Science, Guangdong Medical University Affiliated Hospital, Zhanjiang 524001, China; E-Mail: jingjingzhang12@163.com; 5 Department of Pharmacy, Sichuan Academy of Medical Science & Sichuan Provincial People’s Hospital, Chengdu 610072, China; 6 Center for Perinatal Research, Nationwide Children’s Hospital, Columbus, OH 43205, USA

**Keywords:** islet regeneration, partial pancreatectomy, mangiferin, cell cycle, β-cell

## Abstract

Mangiferin, a xanthonoid found in plants including mangoes and iris unguicularis, was suggested in previous studies to have anti-hyperglycemic function, though the underlying mechanisms are largely unknown. This study was designed to determine the therapeutic effect of mangiferin by the regeneration of β-cells in mice following 70% partial pancreatectomy (PPx), and to explore the mechanisms of mangiferin-induced β-cell proliferation. For this purpose, adult C57BL/6J mice after 7–14 days post-PPx, or a sham operation were subjected to mangiferin (30 and 90 mg/kg body weight) or control solvent injection. Mangiferin-treated mice exhibited an improved glycemia and glucose tolerance, increased serum insulin levels, enhanced β-cell hyperplasia, elevated β-cell proliferation and reduced β-cell apoptosis. Further dissection at the molecular level showed several key regulators of cell cycle, such as *cyclin D1*, *D2* and cyclin-dependent kinase 4 (*Cdk4*) were significantly up-regulated in mangiferin-treated mice. In addition, critical genes related to β-cell regeneration, such as pancreatic and duodenal homeobox 1 (*PDX-1*), neurogenin 3 (*Ngn3*), glucose transporter 2 (*GLUT-2*), Forkhead box protein O1 (*Foxo-1*), and glucokinase (*GCK*), were found to be promoted by mangiferin at both the mRNA and protein expression level. Thus, mangiferin administration markedly facilitates β-cell proliferation and islet regeneration, likely by regulating essential genes in the cell cycle and the process of islet regeneration. These effects therefore suggest that mangiferin bears a therapeutic potential in preventing and/or treating the diabetes.

## 1. Introduction

In both type 1 and type 2 diabetes, hyperglycemia, which partly results from either loss of β-cell mass or insulin resistance, leads to considerable morbidity in the world population [1,2]. Although islet transplantation has been widely used as a regular clinical surgery for diabetic patients, it is still restricted due to the limit of appropriate organ donors. However, under appropriate circumstances, substantial compensatory capacity can be triggered by β-cells in response to physiological and pathophysiological changes. Therefore, a promising alternative to islet transplantation is stimulation of the regeneration of endogenous β-cells, which replaces the deficiency of β-cell mass in diabetic patients. In fact, a classic model of regeneration in rodents is partial pancreatectomy (PPx), which is followed by replication of pre-existing differentiated cells, hypertrophy of β-cells and the differentiation of whole new lobes of pancreas [3]. As for children and juveniles with dysfunctional immune systems, the islet β-cells are attacked by their own immune systems that would result in islet beta cells killing in a very short period of time [4,5]. This type I diabetes (IDDM) usually presents in teenagers, and relies on supplement of exogenous insulin [6]. Our PPx surgery is an artificial method that leads to acute loss of islet beta cells in a short period time; therefore, our PPx model is an effective model for type I diabetes resulting from acute loss of islet beta cells in a short time. It has been proved that PPx, a classical model of regeneration in rodents, is followed by replication of pre-existing differentiated cells, hypertrophy of β-cells and the differentiation of whole new lobes of the pancreas [3]. Studies by Xu *et al.* [7] have demonstrated that partial duct ligation could induce neogenesis. Therefore we established partial pancreatectomized mice to study whether mangiferin therapy could induce islet regeneration *in vivo*, which would be beneficial to young diabetic patients with IDDM.

Previous studies demonstrated that mangiferin, a traditional Chinese medicine isolated from the leaves of mango (*Mangiferina indica*) possesses many beneficial biological activities, including antioxidant [8], antitumor [9], antiviral [9] and immunomodulatory activities [10]. Mangiferin was also reported to have anti-diabetic activities by obviously lowering levels of fasting plasma glucose in STZ-induced diabetic rats [11] Treatment with mangiferin has significantly improved the activity of cardiac and renal antioxidant enzymes, and decreased levels of glycosylated haemoglobin and CPK in STZ-induced diabetic rats [11,12]. These anti-diabetic effects of mangiferin might be due to stimulation of peripheral glucose utilization, enhancement of glycolytic and glycogenic processes, and/or a glycemia reduction through the inhibition of glucose intake [13,14]. Interestingly, in glucose-loaded normal rats, mangiferin also induces significant improvement of oral glucose tolerance without alteration of basal plasma glucose level [11]. For example, in a type 2 diabetes animal model, oral administration of mangiferin reduces the baseline glucose level by more than half [15]. Besides, administration of 30 mg/kg mangiferin followed by 30 min daily exercise decreases blood cholesterol by 40% and triglyceride levels by 70% [16]. Nevertheless, very few studies have been pursued regarding mangiferin’s effects on type 1 diabetes, and their underlying mechanisms are also not known.

Our study here reports for the first time that mice pancreas regeneration after PPx was significantly enhanced by mangiferin treatment. Further, this pancreas regeneration was accompanied with increased body weight, and controlled blood glucose levels, increased serum insulin concentrations, and increased β-cell mass. Importantly, markedly elevated BrdU-positive β-cells indicated that β-cell proliferation is effectively enhanced by mangiferin, while on the other hand apoptosis of β-cells is diminished. Furthermore, molecular analyses performed by immunoblotting and real-time PCR showed that mangiferin modulates islet β-cell proliferation by regulating its cell cycle and essential proteins related to islets regeneration and glucose metabolism, such as cyclinD1, cyclinD2 and Cdk4, p27, GLUT-2, GCK, PDX-1, Foxo-1and Ngn3.

## 2. Results

### 2.1. Mangiferin Induces an Increased Insulin Release to Promote Glucose Homeostasis

**Figure 1.**
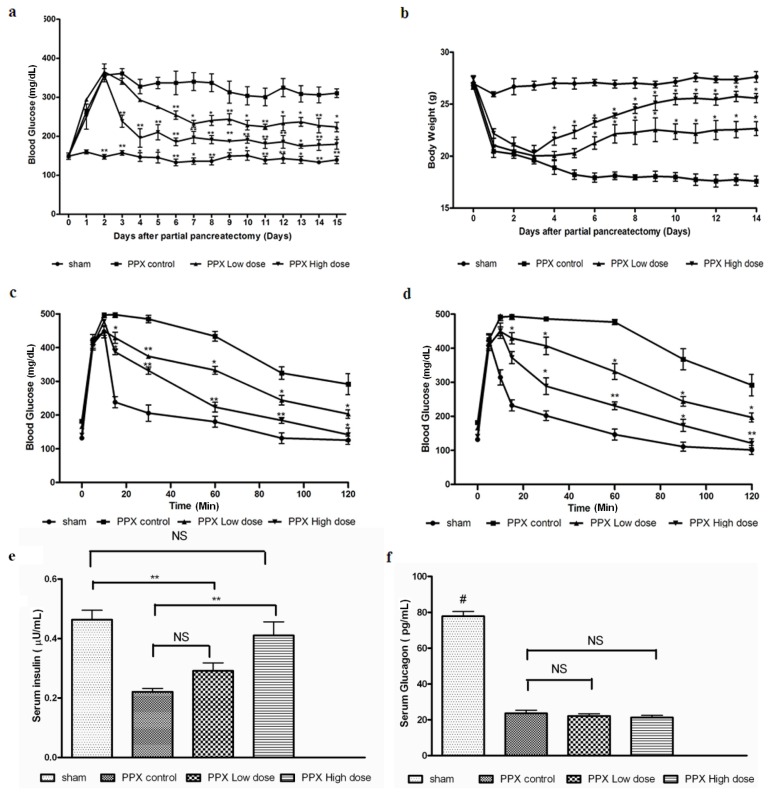
Mangiferin induces glucose metabolic changes in mice after pancreatectomy. (**a**) Comparisons of fasting blood glucose concentrations 14 days post surgery. Day 0 represented as the day mice received surgery, and all mice treated with different dosages of mangiferin from day 1; (**b**) The variation of body weight after a 14-day mangiferin treatment. Day 0 represented as the day mice received surgery, and all mice treated with different dosages of mangiferin from day 1; (**c**) Intravenous glucose tolerance test (IVGTT) on day 7 post surgery; (**d**) IVGTT on day 14 post surgery; (**e**) Fasting insulin and glucagon levels on day 14; and (**f**) Glucagon levels on day 14. *n* = 10 in each group. *, *p* < 0.05 and **, *p* < 0.01 *vs.* partial pancreatectomy (PPx) control at the same time point by two-way ANOVA for repeated measures. # *p* < 0.05 *vs.* all other groups at the same time point by one-way ANOVA. Data are means ± SEM. NS: not significant.

Previous reports have shown that mangiferin (30–90 mg/kg) decreases plasma glucose in a dose-dependent manner. Both high-dose (90 mg/kg body weight) and low-dose (30 mg/kg body weight) of mangiferin were administrated continuously for 2 weeks for the pancreatic regeneration studies. Daily fasting blood samples from all 4 groups of mice were collected and analyzed for fasting glucose levels over a period of 7- and 14-day post-surgery. Fasting blood glucose concentrations were lower than 300 mg/dL after 14 days of therapy in either low-dose or high-dose mangiferin treated mice (Figure 1a). In contrast, mice treated with control solvent DMSO displayed fasting blood glucose concentrations consistently >300 mg/dL. Body weight of mice treated with control solvent also decreased significantly after the surgery, mainly due to sudden loss of pancreas and impaired digestive ability. However, in both mangiferin treated groups, a comparative stable body weight was not significantly different from the sham operated group (*p* > 0.05) (Figure 1b).

After 7 and 14 days post surgery, glucose tolerance tests were performed, and the corresponding results are shown in Figure 1c,d. Fasting insulin levels in both groups were also significantly increased (Figure 1e), while the circulating glucagon levels were not obviously increased (Figure 1f), compared with the PPx control group. In summary, mangiferin treatment improves glucose tolerance of PPx operated mice effectively, while high dose mangiferin exerted more potent effects than the low dose group.

### 2.2. Mangiferin Treatment Enhances Proliferation of β-Cells and Duct Cells

**Figure 2.**
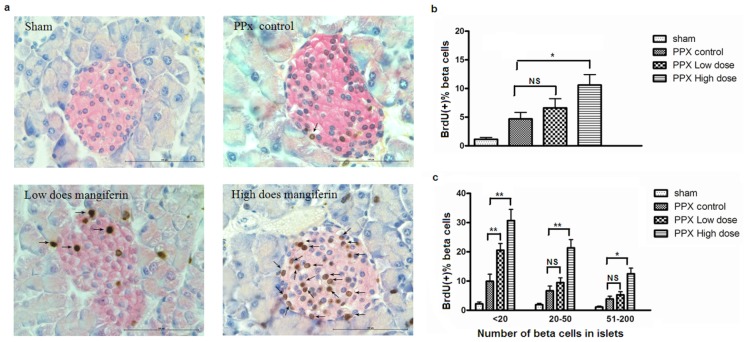

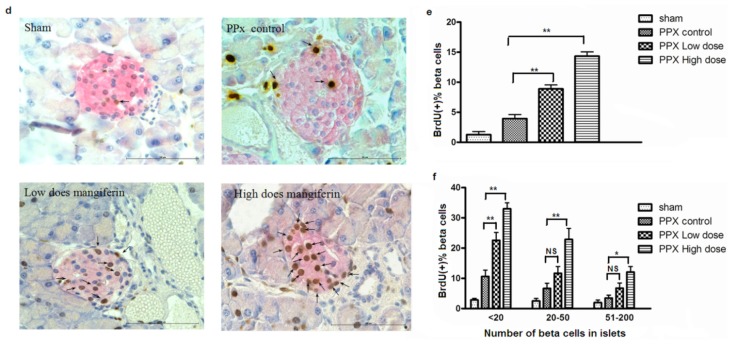
Mangiferin induces islet regeneration. (**a**) Representative pictures of immunohistochemistry staining of insulin-positive cells (red) and bromodeoxyuridine (BrdU)-labeled cells (brown) of different groups after 7 days treatment. Arrows point to the BrdU-labeled cells. Scale bar represents 100 μm; (**b**) BrdU-positive percentage of insulin-positive β-cells from different groups on day 7. At least 10 islets with more than 1000 β-cells were counted per mouse (*n* = 8). *, *p* < 0.05 and **, *p* < 0.01 *vs.* PPx control group at the same time point by one-way analysis of variance; (**c**) Assessment of the distribution of proliferated β-cells in different islet size on day 7. Data are means ± SEM of 3 independent experiments and statistical significance. *, *p* < 0.05 and **, *p* < 0.01 *vs.* PPx controls group by two-way analysis of variance; (**d**) Representative pictures of immunohistochemistry staining of insulin-positive cells (red) and bromodeoxyuridine (BrdU)-labeled cells (brown) of different groups after 14 days treatment. Arrows point to the BrdU-labeled cells. Scale bar represents 100 μm; (**e**) BrdU-positive percentage of insulin-positive β-cells from different groups on day 14. At least 10 islets with more than 1000 β-cells were counted per mouse (*n* = 8). **, *p* < 0.01 *vs.* PPx controls group by one-way ANOVA; and (**f**) Assessment of the distribution of proliferated β-cell in different islet size; *n* = 8 for each group and each time point. Data are means ± SEM of 3 independent experiments and statistical significance *, *p* < 0.05 and **, *p* < 0.01 *vs.* PPx controls group by two-way analysis of variance. NS: not significant.

**Figure 3.**
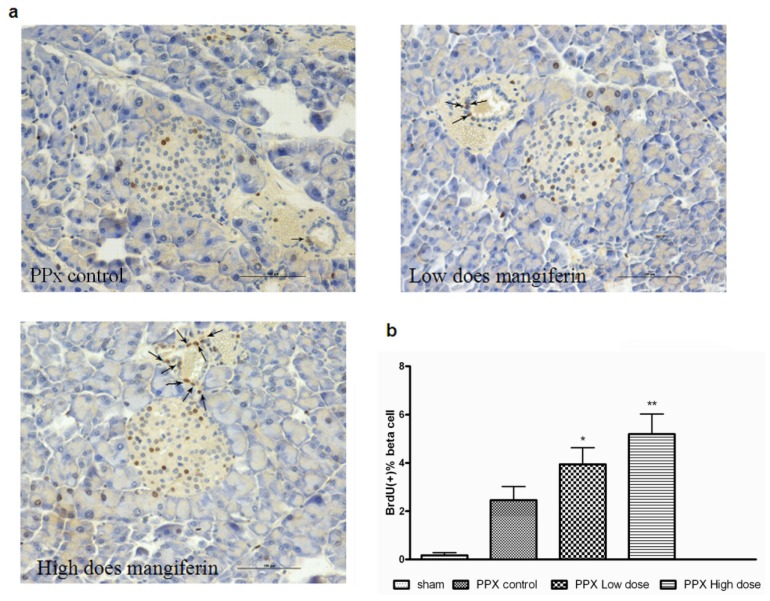
Mangiferin promotes proliferation of duct cells. (**a**) Representative pictures of immunohistochemistry staining of insulin-positive cells (red) and BrdU-labeled cells (brown). Arrows point to the BrdU-labeled duct cells. Scale bar represents 100 μm; and (**b**) BrdU-positive percentage of duct cells. *n* = 8 for each group.* *p* < 0.05 and **, *p* < 0.01 *vs.* PPx control group by one-way ANOVA; Data are means ± SEM. PPx, partial pancreatectomy.

To investigate whether mangiferin was implicated in β-cell proliferation after a sudden loss of pancreatic β-cells by pancreatectomy, islet β-cells in active proliferation were detected by continuous BrdU incorporation and subsequently quantification. Interestingly, pancreas sections, which were double stained by BrdU (brown) and insulin (red), exhibited a remarkable increase of BrdU-positive cells in mice by either 90 or 30 mg/kg mangiferin treatment (Figure 2a,d; arrows point to the BrdU labeled β-cells). The significant increase of β-cell regeneration was confirmed by quantification analyses (Figure 2b,e). Furthermore, to investigate whether islet size would impact on β-cell proliferation in response to mangiferin treatment, islets were stratified by the similar number of β-cells in islet cross-section. It demonstrated that mangiferin treatment up-regulated β-cell proliferation in all various sizes of islet (Figure 2c,f), though it has been reported [17] that small islets (<20 β-cells) have higher rates of proliferation than larger islets. Since proliferation/neogenesis of both β-cells and duct cells contributed to β-cell hyperplasia after PPx, we also calculated BrdU-positive duct cells (Figure 3a). Interestingly, a more prominent increase of duct cell proliferation was detected in the mangiferin treatment group (Figure 3b). Therefore, mangiferin contributes to proliferation of both β-cells and duct cells.

### 2.3. β-Cell Apoptosis Is Inhibited by Mangiferin

**Figure 4.**
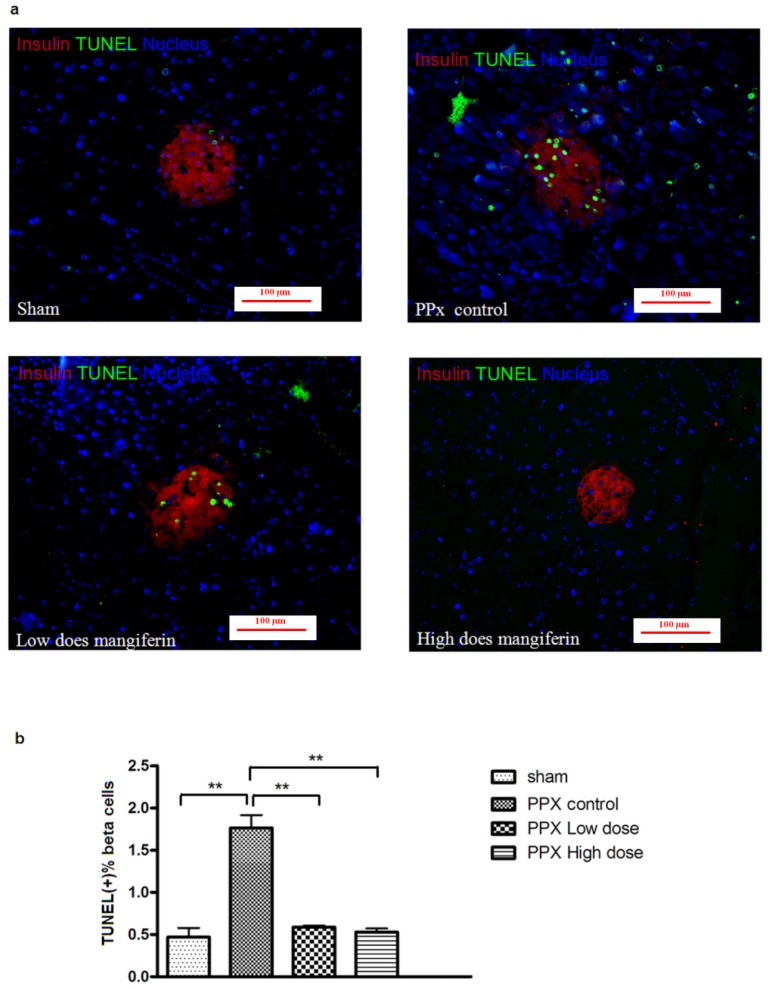
Mangiferin inhibits β-cell apoptosis. (**a**) Representative pictures of immunofluorescent staining of insulin (red) and TUNEL (green) of mangiferin treated or PPx mice, on day 14. At least 10 islets with more than 1000 β-cells were counted per mouse. All graphs show means ± SEM from at least 2 independent experiments. Scale bar represents 100 μm; and (**b**) TUNEL-positive percentage of different groups on day 14. At least 10 islets with more than 1000 β-cells were counted per mouse; *n* = 8 for each group and each time point. All graphs show means ± SEM from three independent experiments. **, *p* < 0.01 *vs.* PPx controls group by one-way ANOVA.

To assess whether mangiferin treatment would prevent β-cells from apoptosis, a TUNEL staining of remnant pancreas post surgery was performed (Figure 4a). As shown at Figure 4b, both doses of mangiferin treatment resulted in a lower level of β-cell apoptosis than the control group. As expected, comparatively higher β-cell apoptosis was found in low dose mangiferin groups. Therefore, rate change of β-cell apoptosis might also contribute to the increased insulin secretion *in vivo*, along with enhanced β-cell proliferation.

### 2.4. Mangiferin Increases β-Cell Mass

**Figure 5.**
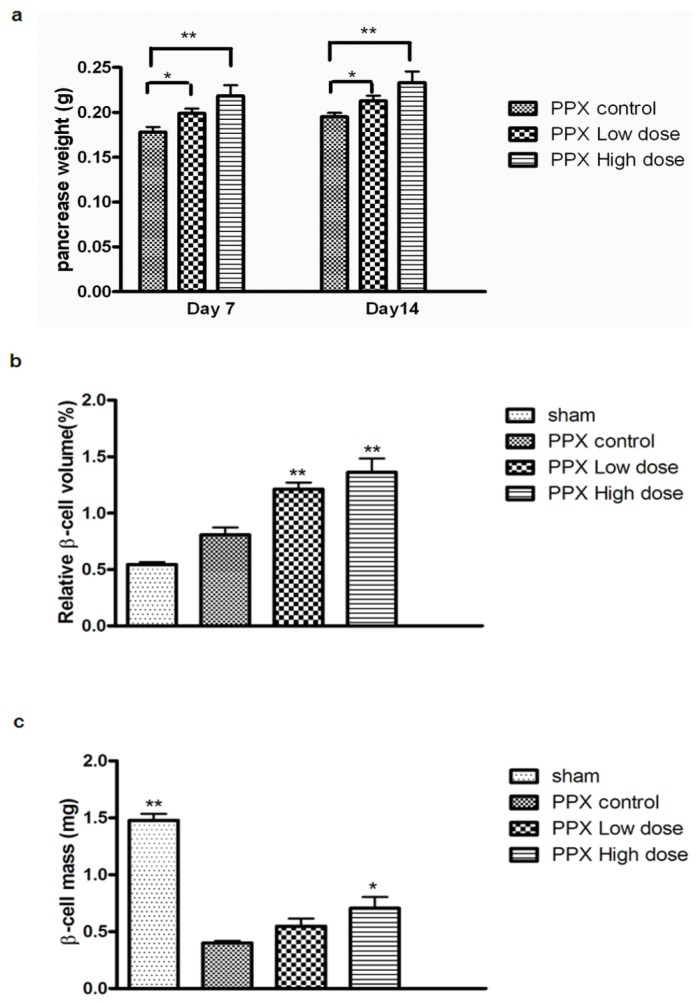
Mangiferin induces β-cell hyperplasia. (**a**) Comparisons of remnant pancreas weight; (**b**) Relative β-cell volume by point counting; and (**c**) β-cell mass calculated by relative β-cell volume and total weight of remnant pancreas. Two or three slides (200 μm apart) from the broadest pancreatic sections were analyzed for β-cell mass measurement. (*n* > 6 for each group). All graphs show means ± SEM from three independent experiments. (*, *p* < 0.05 and **, *p* < 0.01 compared with PPx controls group).

To investigate whether mangiferin therapy would actually induce islet hyperplasia, the relative β-cell volume and mass in remnant pancreas were subsequently quantificated. Further, remnant pancreas weight was significantly increased in mangiferin-treated mice (Figure 5a). We found that compared with PPx control or sham group, relative β-cell volume in the mangiferin-treated group was substantially increased (Figure 5b). Thus, it can be concluded that absolute β-cell mass in remnant pancreas was significantly upregulated through mangiferin treatment, which is consistent with the increase of β-cell proliferation and the reduction of β-cell apoptosis (Figure 5c).

### 2.5. Mangiferin Up-Regulates both Expression and Activity of Cell Cycle Regulators

**Figure 6.**
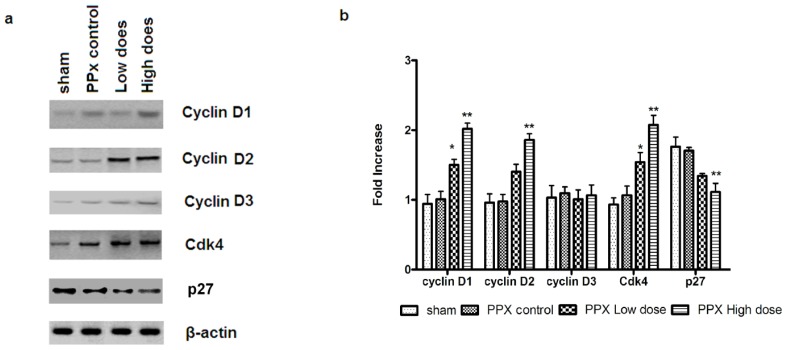
Mangiferin modulates the mRNA and protein level of β-cell cycle regulators. Immunoblotting (**a**) and real time reverse transcription polymerase chain reaction (RT-PCR) (**b**) analyses of *cyclin D1*, *cyclin D2*, *cyclin D3*, *Cdk4*, *p27* (*n* = 10 for each group, *, *p* < 0.05, and **, *p* <0.01, *vs.* PPx controls group by two-way analysis of variance).

CyclinD-Cdk4 complex plays an essential role in β-cell cycle progression. To investigate the involvement of mangiferin in β-cell cycle regulation, the expression of a few key cell-cycle regulators such as cyclin D1, cyclin D2, cyclin D3, Cdk4, p27 and were measured, at both protein and mRNA level. As shown in Figure 6a,b, increase in Cdk4 mRNA and protein expressions were observed in the mangiferin-treated group and the mRNA and protein levels of cyclin D1 and cyclin D2, though not cyclin D3, were also increased. Further, p27 was decreased at both the mRNA and protein levels.

**Figure 7.**
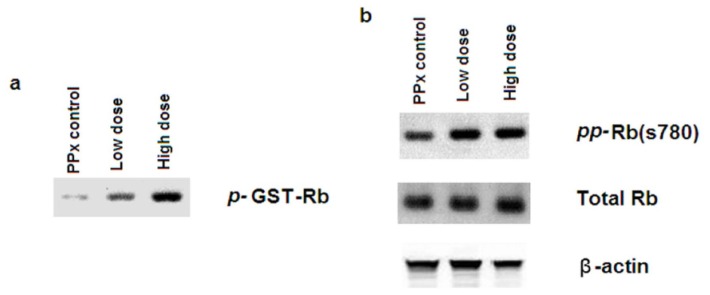
Mangiferin up-regulates Cdk4 enzymatic activity. (**a**) *In vitro* Cdk4 kinase activity in islets from mangiferin-treated and untreated control mice. Islets from remnant pancreas of 50 mice were pooled, with recombinant GST-Rb; and (**b**) Immunoblotting for hyperphosphorylated Rb at Ser780 (**upper** panel). Total Rb (**middel** panel) and β-actin (**bottom** panel) in islet lysates were showed. The data represent one out of three independent experiments that gave similar results.

In addition, the enzymatic activity of Cdk4 was measured through an *in vitro* kinase assay with immuno-purified Cdk4 proteins from mice islets and by using the recombinant GST-Rb (amino acids 769–921) as substrate that was previously described [18,19]. Rb itself also plays a fundamental role in cell cycle progression through its association with the E2F family of transcription factors, and it is an important substrate of the cyclinD1/Cdk4 complex. In this study, the incorporation of radioactive phosphate to this substrate is proportional to Cdk4 activity in the immunoprecipitates. As shown in Figure 7a, phosphorylation of recombinant GST-Rb was significantly increased in islet lysates from the mangiferin-treated mice group, indicating the overall enzymatic activity of Cdk4 was promoted by mangiferin treatment. Furthermore, immunoblotting of phosho-Rb (Ser780) was performed by isolated islet samples, and an increase in Rb phosphorylation was prominent in islets from mangiferin-treated mice (Figure 7b). These results indicate that mangiferin promotes the activity of the cyclin D1/Cdk4 complex that is important in β-cell proliferation. Therefore, mangiferin may initiate β-cell proliferation through regulating the expression and activity of related cell cycle regulators.

### 2.6. Mangiferin Modulates the Expression of β-Cell-Specific Genes

**Figure 8.**
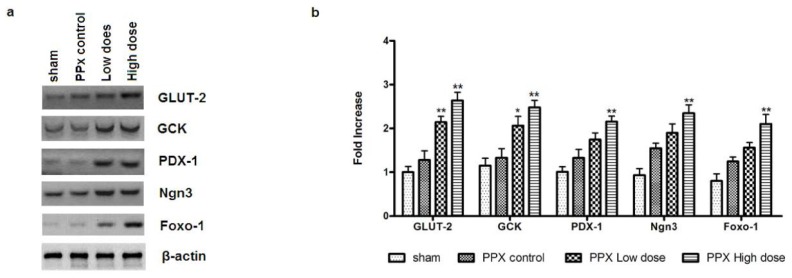
Expression change of important proteins for β-cell regeneration and function by mangiferin. (**a**) Representative immunoblots for islets isolated from remnant pancreas, by indicated antibodies. *n* = 6, for PPx control mice; *n* = 8, for mangiferin-treated mice. Protein expression of GLUT-2, GCK, PDX-1, Ngn3, and Foxo-1 were examined in cell lysates. Protein bands shown are representative from more than 3 independent experiments with similar results; and (**b**) Real time RT-PCR analyses of *GLUT-2*, *GCK*, *PDX-1*, *Ngn3*, *Foxo-1. n* = 10 for each group, All data are means ± SEM. * *p* < 0.05, and ** *p* < 0.01 *vs.* PPx control by two-way analysis of variance. The data represent one out of three independent experiments that gave similar results.

Several β-cell-specific genes, including GCK, GLUT-2, PDX-1, Ngn3 and Foxo-1, are critical for islet regeneration and β-cell function. We assumed expression changes of these genes might contribute to the mangiferin-induced reduction of fasting blood glucose concentration. For this purpose, Real time PCR and immunoblotting assays were performed to assess such changes. As shown at Figure 8, a significant increase in both protein and mRNA expressions of GLUT-2, GCK and Foxo-1 were detected in islets from mangiferin-treated mice (Figure 8). Additionally, recent studies showed that Cdk4 promotes β-cell replication by activating *PDX-1* and *Ngn3* genes [20,21], and *Ngn3* is also involved in β-cell neogenesis. Actually, we found a remarkable increase of both *PDX-1* and *Ngn3* expressions at either the protein or mRNA level in islets form mangiferin-treated mice (Figure 8).

### 2.7. Islet Viability Analysis

**Figure 9.**
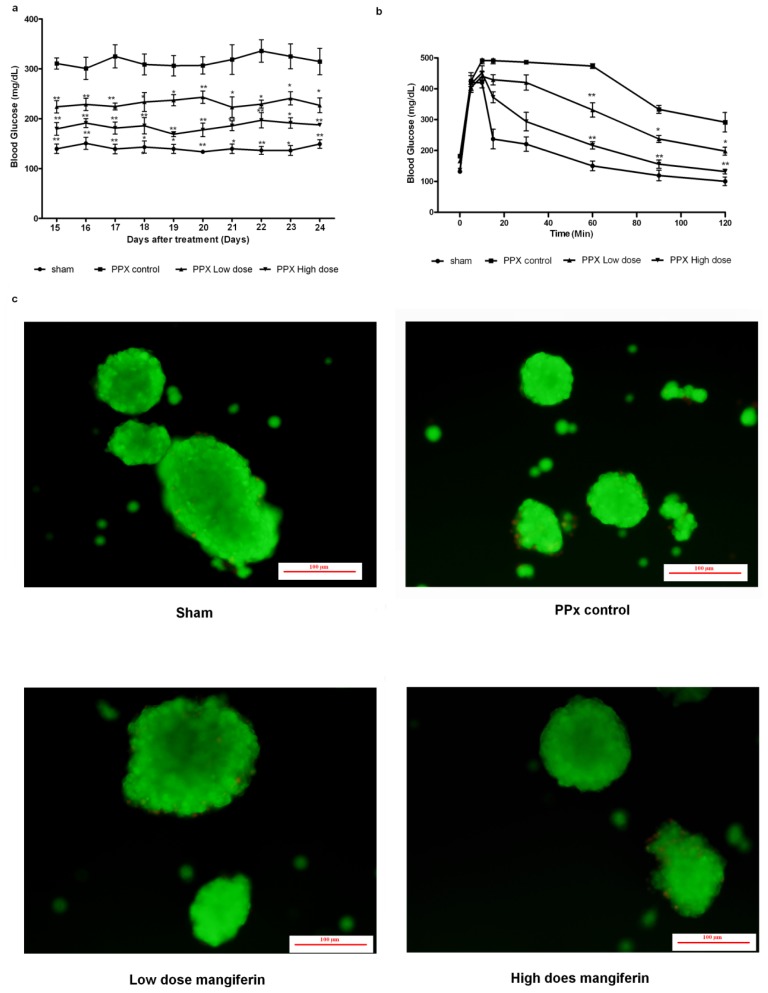
Mangiferin-induced irreversible islet β-cell regeneration (**a**) Comparisons of fasting blood glucose concentrations at the 10th day after mangiferin treatment; (**b**) IVGTT on day 10 after 14-day treatment of mangiferin or DMSO; and (**c**) Islet viability was preserved after 10 days feeding. FDA was represented as viable cells with green fluorescence, while nonviable cells were stained with PI and exhibited as red fluorescence. Scale bar represents 100 μm. All data are means ± SEM. * *p* < 0.05, and ** *p* < 0.01 *vs.* PPx control by two-way analysis of variance.

Another 40 PPx mice were randomly devided into four groups, and after treatment with mangiferin or DMSO for 14 days, were kept for another 10 days feeding without any treatment. Blood glucose concentrations were measured every day after treatment, and Figure 9a revealed that after feeding for 10 days without mangiferin treatment, blood glucose concentrations were steadily maintained below 300 mg/dL. At the 10th day after feeding, glucose tolerance tests were performed, and corresponding results also revealed that mangiferin treatment for 14 days could effectively maintain glucose tolerance without reversable effects.

In addition, in order to demonstrate whether mangiferin would result in pancreatic abnormalities, we performed FDA/PI (fluorescein diacetate/propidium iodide) standard staining. Quantitative comparisons of the areas of the green (FDA, represented as viable cells) and red (PI, represented as death cells) regions suggested that percent viability of the Sham group, control group, low dosage of mangiferin and high dosage of mangiferin were 98%, 96%, 97% and 98%, respectively.

## 3. Discussion

Diabetes mellitus, a complex syndrome, is featured by insulin deficiency or resistance. It mainly results from the inadequate transportation of glucose from the vasculature into fat and muscle that finally leads to hyperglycaemia. Diabetic patients exhibit altered glucose, fat as well as protein metabolism. Further, this dysfunction substantially changes the cellular microenvironment in many different tissue types, leading to “diabetic complications” including microvascular complications such as diabetic nephropathy (DN), neuropathy and retinopathy, along with macrovascular complications including accelerated atherosclerosis causing ischaemic heart and cerebrovascular disease [22].

There are two major forms of diabetes, type 1 diabetes mellitus (T1DM) and type 2 diabetes mellitus (T2DM). The former occurs when the insulin-producing β-cells in the pancreas are destroyed, typically through an autoimmune disease [23,24]. The latter is caused by a resistance to insulin combined with a failure to produce sufficient insulin [25,26]. Both T1DM and T2DM would finally converge to common symptoms, such as glucose intolerance and hyperglycaemia. Hyperglycemia both in T1DM and T2DM mainly results from the loss of β-cell mass that would lead to considerable morbidity in the world population. Recently, an alternative and promising strategy in development is to stimulate the regeneration of endogenous β-cells to replace the deficit in β-cell mass in diabetic patients [27–29].

Previous reports have demonstrated that several TCM (traditional Chinese medicine) extracts could exert antidiabetic effects. Among them, mangiferin was reported to slow fasting plasma glucose level significantly at different time intervals, and showed other antidiabetic activities in STZ-diabetic rats [11]. Treatment of mangiferin effectively improved the dysfunctions of antioxidant enzyme activities in cardiac and renal systems, decreasing levels of glycosylated haemoglobin and CPK in STZ-induced diabetic rats [11,12]. This effect is considered to be a stimulation of peripheral glucose utilization, the enhancement of glycolytic and glycogenic processes, and/or a glycaemia reduction through the inhibition of glucose intake [13,14]. Mangiferin also improved oral glucose tolerance without alteration of basal plasma glucose levels in glucose-loaded normal rats [11].

For the first time, our study reported here that the mice pancreas regeneration after PPx was significantly enhanced by mangiferin treatment. It was accompanied by increased body weight, controlled blood glucose levels, serum insulin concentrations, and β-cell mass. Importantly, markedly elevated BrdU-positive β-cells indicated that β-cell proliferation is effectively enhanced by mangiferin, while on the other hand apoptosis of β-cells was diminished. The pancreas has both the exocrine and the endocrine activities. The function of exocrine is to secrete digestive enzymes, while the latter plays important roles in the regulation of blood sugar. Both the exocrine and endocrine tissue were taken away after pancreatectomy, therefore the digestive enzymes were correspondingly decreased, which can not meet the body requirement. As a feedback, it will ultimately result in the proliferation of exocrine. Though the exocrine proliferation still seems quite high, the activity of those digestive enzymes would not affect the level of blood glucose. This could be demonstrated by the levels of blood glucose and IVGTT results. It is speculated that exocrine proliferation might make up the lack of digestive enzymes.

Mangiferin could play a significant role in the adaptive expansion and regeneration of β-cell mass after PPx. Previous reports demonstrated that expansion of β-cell mass was linked to proteins implicated in cell-cycle progression [30,31]. Many reports have confirmed that cyclin D2 was crucial for controlling postnatal β-cell expansion [32]. Mangiferin modulation of islet β-cell proliferation might occur through regulating its cell cycle and essential proteins related to islet regeneration and glucose metabolism, such as cyclinD1, cyclinD2, Cdk4, p27, and GLUT-2, GCK, PDX-1, Foxo-1 and Ngn3. In fact, both the mRNA and protein expressions of cyclinD1 and cyclinD2 were significantly increased in mice treated with mangiferin.

Intriguingly, mangiferin upregulated Cdk4 in both expression and overall enzymatic activity to phosphorylate Rb. It has been documented that Cdk4 coimmunoprecipitates with p16 [33], and the expression level of p16 increase dramatically in senescent cells [34]. It might be that the delivery of magniferin could induce cell senescence. However, as a potential Cdk4 inhibitor, we did not observe significant decreases in the expression level of Cdk4 in the groups of mice whose p16 expression levels appear to be increased. We assume that Cdk4 and other cell cycle regulators might be regulated with proteins other than p16, because p16 is not the only protein which could regulate all the cell cycle proteins. There might also be a feedback regulation from senescence and proliferation after partial pancreatectomy, especially after the delivery of mangiferin.

Cdk4 has previously been suggested to be necessary to maintain postnatal β-cell proliferation [35,36], and it also plays an important role in promoting β-cell development, by directing E2F1-mediated activation of Ngn3 and increasing the pool of endocrine precursors [20]. In addition, one report has shown that activation of *Ngn3* gene contributed to the differentiation of adult progenitors and the glucose responsiveness of differentiated β-cells. Moreover, upstream signaling pathways such as PDX-1 are crucial in the regulation of β-cell regeneration [37,38], and may be correlated with increased expression and activity of the cyclinD-Cdk4 complex. As expected, these critical regulators of β-cell regeneration and function were regulated by mangiferin. Beyound this, we also observed that duct cells were proliferated after mangiferin treatment, which was concomitant with increased expression of Ngn3, PDX-1 and Foxo-1.

Fianlly, in order to further detect whether mangiferin reversibly induced islet β-cell proliferation, or whether mangiferin resulted in any pancreatic abnormalities, blood glucose and glucose tolerance tests, as well as viability analysis were performed. All of the results supported that mangiferin treatment could effectively maintain blood glucose stabilization and glucose tolerance without reversable effects and would not lead to pancreatic abnormalities.

## 4. Materials and Methods

### 4.1. Study Design

Eight-week-old male C57BL/6J mice (*n* = 120) were randomly divided into four groups:

Sham group (*n* = 30): Mice received sham operation. PPx control group (*n* = 30): Mice were administered DMSO after PPx operation. High dose mangiferin group (*n* = 30): Mice were administered 90 mg/kg mangiferin after PPx operation [15]. Low dose mangiferin group (*n* = 30): Mice were administered 30 mg/kg mangiferin after PPx operation [16].

Mangiferin was isolated and maintained by our lab. For the mangiferin group, all the mice were treated with mangiferin dissolved in DMSO for 14 consecutive days. Then all the mice were sacrificed, and the remnant pancreases were harvested for the study of drug efficacy. All animals were handled according to the guidelines of the Sichuan University Medical Center Institutional Animal Care and Use Committee. The local ethics committees approved this study.

### 4.2. Animal Surgery

Animals were fasted for 12 h before operation, and 5 h after the operation animals were allowed free access to standard diet and water. Mice were anesthetized with 10% Chloral hydrate (Sigma, Bellefont, PA, USA) and the abdomen were opened through an upper midline incision. The spleen and the entire splenic portion of the pancreas were surgically removed, but the mesenteric pancreas between the portal vein and duodenum was left intact. This operation resulted in a 70% pancreatectomy, which was confirmed by weighting the removed and remnant portions. The sham operation was performed by removing the spleen while leaving the pancreas intact. The incision was closed using 5-0 silk sutures. The proliferating cells were labeled with BrdU (0.8 mg/mL; Sigma) in drinking water, and this labeling as well as treatment of mangiferin continued for 14 days from the day after operation.

### 4.3. Biochemical Measurements

After fasting for 8 h, blood glucose concentrations were measured by a Surestep Blood Glucose meter (Lifescan, Milpitas, CA, USA), and any blood glucose concentrations higher than 500 mg/dL were not included. IVGTTs were performed by tail vein injection of D-glucose (1 g/kg; Sigma) on days 7 and 14. After fasting for 8 h, plasma insulin concentrations were determined by using an ultrasensitive mouse insulin ELISA kit (Alpco, Salem, NH, USA), following the manufacturer’s instructions. After fasting for 8 h, plasma glucagon levels were determined by using a glucagon ELISA kit (Wako Pure Chemical Industries, Osaka, Japan).

### 4.4. Immunoblotting

The pancreatic duct was injected with 1 mL collagenase P (Roche, Indianapolis, IN, USA). Islets were isolated as described, handpicked, washed once in phosphate buffered saline and frozen at −80 °C [39,40]. For Western blots, islets were lysed and the protein lysates were centrifuged at 12,000 rpm for 30 min. Supernatants were collected and loaded in a sodium dodecylsulfate PAGE gel (Bio-Rad, Hercules, CA, USA) for electrophoresis. After electrophoresis, proteins were transferred onto polyvinylidene fluoride membrane (Bio-Rad) in transfer buffer. Membranes were blocked with 5% non-fat dry milk in Tris-buffered saline for one hour. Blots were incubated overnight at 4 °C with primary antibodies at the dilutions recommended by the manufacturer, followed by incubating with horseradish peroxidase-conjugated secondary antibody (Zymed, San Francisco, CA, USA), and then visualized by enhanced chemiluminescence reagents (Amersham, Piscataway, MA, USA) [17].

### 4.5. Quantitative Reverse Transcription Polymerase Chain Reaction Analysis

RNA was extracted from islet using the RNeasy micro kit (Qiagen, Valencia, CA, USA) and reverse transcribed (1 μg total RNA, 1 μL random primer (50 μmol/L; Applied Biosystems, Foster City, CA, USA), 1× reverse transcriptase buffer and 10 units reverse transcriptase (Promega, Madison, WI, USA) in a total volume of 20 μL). The RNA and primer were heated to 72 °C and slowly cooled before reverse transcription at 42 °C for one hour. The room temperature reaction was then diluted to 100 μL with RNase-free water. For real-time PCR analysis, 2.5% of the total room temperature reaction was used as input for PCR using SYBR Green Master Mix (Applied Biosystems) on an ABI 7900 Real-Time PCR System (Applied Biosystems, Foster City, CA, USA). Primers for cyclin D1, cyclin D2, cyclin D3, Cdk4, GLUT-2, GCK, Foxo-1, PDX-1 and Ngn3 were previously described [17,41]. For analysis of Ngn3 mRNA abundance, RNA was extracted from the remnant pancreas, and for analysis of other genes, RNA was extracted from islets [41].

### 4.6. Cdk4 Kinase Activity

Cdk4 kinase assays were carried out based on previously described protocols [41]. Pooled islets from remnant pancreas of 40 mice were lysed in lysis buffer, and protein concentration was determined using a BCA Protein Assay kit (Beyotime, Suzhou, China). For each sample, 300 μg of total protein was immunoprecipitated using 1 μg anti-Cdk4 (Abcam, Cambridge, MA, USA) and 50 μL protein G Sepharose beads (Sigma). The final kinase reaction was carried out in 50 mmol/L HEPES, pH 7.5, 10 mmol/L MgCl_2_, 1 mmol/L dithiothreitol, 2.5 mmol/L EGTA, 10 mmol/L glycerophosphate, 0.1 mmol/L Na_2_VO_4_, 1 mmol/L NaF, 5 μmol/L ATP, 6 mCi per reaction of [γ-^32^P]ATP (Amersham) and GST-Rb 769-921 (Santa Cruz Biotechnology, Santa Cruz, CA, USA). Samples were incubated at 30 °C for 30 min and separated by PAGE. The amount of ^32^P-labeled GST-Rb was evaluated by autoradiography and quantified using PhosphorImager and ImageQuant (Molecular Dynamics, Sunnyvale, CA, USA) analysis.

### 4.7. Histology Staining

Tissues were fixed in 10% formalin for 24 h at room temperature, dehydrated, embedded in paraffin and sectioned. For immunohistochemical staining, rat anti-BrdU antibody (Abcam), guinea-pig anti-insulin antibody (Dako, Glostrup, Denmark) was used. Primary antibodies were detected with corresponding secondary antibodies (Jackson Immunoresearch, West Grove, PA, USA). BrdU(+) β-cells and islets were visualized by DAB (Beyotime) and Fast red (Sigma), respectively. For TUNEL staining, the Promega fluorescence Dead End Kit (Promega, Madison, WI, USA) was used following the manufacturer’s instructions.

Islets were photographed at 200× or 400× by a Nikon 80i microscope (Tokyo, Japan). All photograph and statistical works were performed by two blinded technicians. The number of β-cells, BrdU(+) β-cells and TUNEL(+) β-cells was manually counted, and double-checked with Image Pro Plus 6.3 software (Media Cybernetics, Silver Spring, MD, USA). At least 10 islets with at least 1000 β-cells were counted per mouse, and at least 10 consecutive sections from eight mice per group were stained. The first section and the 10th section were selected to ensure 50 μm distance. Islet diameters were calculated with an intraocular calibrated grid. Islet size was also measured at ×400 image of insulin-stained islets converted to gray scale.

Analysis of relative β-cell volume was performed by point counting morphometry, using a 56-point grid. An average of 10,000 points/mouse was counted. β-cell mass was calculated by multiplying the relative β-cell volume by the total weight of the remnant pancreas.

### 4.8. Islet Viability Detection

After treatment with mangiferin for 14 days, all mice were still fed for 10 days. Then, islet viability was detected by standard FDA/PI method. Briefly, 100 islets were respectively randomly chosen from each groups, and islets were incubated for 30 min in an FDA/PI working solution. And the islets were photographed at ×200 or ×400 by a Nikon 80i microscope (Tokyo, Japan).

### 4.9. Statistical Analysis

All data were expressed as mean values ± SEM from at least three independent experiments. Statistical significance was determined by one-way analysis of variance with Bonferroni correction and two-way analysis.

## 5. Conclusions

Taken together, and consistent with a role in modulating β-cell proliferation and stimulating insulin secretion, mangiferin-treated mice exhibited an improved glycemia and glucose tolerance, increased serum insulin levels, β-cell hyperplasia, elevated β-cell proliferation and reduced β-cell apoptosis. Moreover, mangiferin modulates islet β-cell proliferation by regulating its cell cycle and essential proteins related to islets regeneration and glucose metabolism, such as cyclinD1, cyclinD2 and Cdk4, p27, GLUT-2, GCK, PDX-1, Foxo-1 and Ngn3. However, the effects of magniferin in postprandial insulin levels are still unknown. Overall, these anti-diabetic effects of mangiferin are still preliminary, and solid pre-clinical and clinical examinations should be performed to ensure the safety and efficacy of mangiferin for clinical use. With the gradual clarification of molecular mechanisms for mangiferin-induced anti-diabetic effects, mangiferin might represent a potential anti-diabetic drug for future therapeutics.

## Abbreviations

PPx: partial pancreatectomy
STZ: streptozotocin
CPK: creatine phosphokinase
DMSO: dimethyl sulfoxide
BrdU: bromodeoxyuridine
IVGTTs: Intravenous glucose tolerance tests
ELISA: enzyme-linked immunosorbent assay
real-time PCR: real-time polymerase chain reaction
SD-PAGE: sodium dodecylsulfate polyacrylamide gel electrophoresis
TUNEL: transferase-mediated dUTP nick-end labeling
*Cdk4*: cyclin-dependent kinase 4
*GLUT-2*: glucose transporter 2
*GCK*: glucokinase
*PDX-1*: pancreatic and duodenal homeobox 1
*Ngn3*: neurogenin 3
Foxo-1: Forkhead box protein O1
DN: diabetic nephropathy
T1DM: type 1 diabetes mellitus
T2DM: type 2 diabetes mellitus
TCM: traditional Chinese medicine
